# Sick Nursing in the Sixteenth Century

**Published:** 1887-10-01

**Authors:** 


(*To be continued.*)

### Sick Nursing in the Sixteenth Century.

By a Student of Mediæval Institutions.

It was not until comparatively recently that the direct personal service of the poor was recognised in modern England as a legitimate sphere for the ministration of women. Prior to the Reformation, of course, there had been throughout the country numerous houses of nuns, who were more or less devoted to those branches of charitable work which could be properly undertaken by them; but it is noteworthy that among the many changes which the Church of England experienced in the sixteenth century, no provision was made for recognising the special work of women, or for extending to it that organisation and supervision which are essential to its efficiency. Not until very recent times has what is sometimes called "the religious life" been since then revived among us, and though the attempts to restore community life for men have been as a rule signally unsuccessful, it has been far otherwise in the case of women. The Sisterhoods of the Church of England are at the present day not only numerous, but flourishing; and it may be affirmed, without fear of contradiction, that at no previous period has the work of women been so extensive, so valuable, so efficient as it is to-day. It is a fact full of the brightest hope for humanity at large that there are so many of England's daughters who have given themselves up to the service of the poor and of the sick, whether they have devoted themselves to this life as members of sisterhoods or other bodies, or whether they have simply adopted this vocation without seeking any special religious sanction.

A similar state of things is to be observed in the Church of Rome. Here no such check as that to which allusion has just been made has been experienced; and therefore some of the religious orders of women, which exist in that communion to-day, can trace their descent back into the fourth century, when the primitive Apostolic Order of Deaconesses (see Rom. xvi, 1; Titus ii, 3; 1 Tim. iii, 11) was gradually developing into those more rigid and coherent organisations which became so famous in mediæval times.

The vastness of the forces at the command of the authorities of the Roman Catholic Church is a matter of common observation; and it is true of this particular branch of work as of any other, for there are over 200 separate orders of women which to-day profess obedience to the Pope of Rome. Of course this vast number does not represent so many distinct types of organisation, for between many orders the difference is but very slight. All monastic bodies, whether of men or women, may be roughly divided into two classes: first there are the contemplative orders, the members of which are strictly "enclosed" within the limits of their monastery, where their life is one long mortification of the body (perpetual abstinence from meat being generally enforced, among other rigours), combined with almost unceasing performance of divine worship. The other class is the "active." The members of these orders are engaged in some external work, teaching or tending the sick, the aged poor, orphans, and so on. In these latter orders the "rule" is of course far less rigorous, so as to permit of the members engaging in their various undertakings, though even here the life is by no means luxurious. This distinction between the contemplative and active orders of the Roman Church is the one best appreciated by outsiders; but there is another, and this is drawn between an "order" and a "congregation." An "order," strictly so called, is a society which has received formal Papal confirmation. The members take "solemn" or irrevocable vows, from which it is very rarely that anyone, man or woman, is released. A "congregation" is a society the members of which take vows, which are generally renewable at given periods; and this organisation, moreover, will have received only the approval of a bishop, in whose hands usually rests the power to dispense or release anyone from the vows.

So much by way of preface. Of these 200 and odd societies of women in the Roman Church, by far the most familiar is the congregation of the Sisters of Charity of S. Vincent de Paul, and as it has supplied the model for similar bodies in the English Church, it is deserving of some attention. S. Vincent de Paul, the founder, sprang from the ranks of the peasantry. He was born at Ranquines, in the south of France, in the year 1577, and, having been enabled by wealthy patrons to complete the necessary studies, he received ordination as priest at the age of twenty-three. Soon was he called to "endure hardship as a good soldier," for, during a voyage, he was captured by pirates and sold into slavery. At last he fell into the hands of an apostate Christian, who had married a Turkish woman. S. Vincent succeeded in converting the wife, and through her in reconciling the husband, and in their company he returned to France in 1607. In 1625 he founded his well-known "Prêtres de la Mission"—a society of priests who, without quitting the world entirely, lived in community a much severer and stricter life than was then customary among secular priests. This alone was no small achievement in an age of ecclesiastical luxury and pride. He had before this formed the "Confraternity of Ladies of Charity," who, without taking upon them any sort of vows or religious obligations, visited and relieved the poor. S. Vincent, however, soon found that these ladies living in the world were able indeed to give money, but rarely personal help and service. Often the distribution of alms, that should have been bestowed with accompanying words of consolation and hope, was entrusted to servants who found the work but little to their taste. S. Vincent, therefore, gradually got together a number of girls, chiefly of the peasant class, whose duty it was to see that the alms of the "Ladies of Charity" were properly and effectively distributed: they were, in short, the servants for this special purpose of the "Dames de la Charité;" and with these, as it seems to us, embryonic organisations, S. Vincent was able to accomplish much good, supplementing these societies with a third, the "Assemblée des Dames de la Charité," whose work lay particularly among the hospitals. This last-named body was divided into two classes: one prepared delicacies such as were fitted for invalids, which they distributed among the sick; while it was the business of the second to dispose the recipients to accept the ministrations of the priests. Thus, in 1646, S. Vincent's work stood as follows:—He had established the "Confrérie des Dames de la Charité," with many branches throughout France, and the "Assemblée des Dames de la Charité," both of which bodies had a superior, an assistant, and a treasurer, the director of both being S. Vincent himself. The members were all wealthy, and many of them occupied high positions in the world. Besides these, S. Vincent had brought together a humbler class of women, who were at the disposal of the "Dames de la Charité." At this time they had no distinctive dress, and no formal "rule." Their daily routine was of the most simple description. They rose at four and retired at nine. All the outward religious observances which were demanded of them were that they should hear mass and recite their rosary daily; examine themselves twice a day; meditate for half an hour morning and evening, and recite their prayers together. But, as time went on, S. Vincent determined to give these peasant girls a more definite organisation; and accordingly he obtained the necessary sanction for a code of rules, and in August 1655 formally inaugurated the new community, the Sisters of Charity of S. Vincent de Paul. The constitution has remained unaltered to the present day. The congregation is governed by the Superior-General of the "Prêtres de la Mission," or Lazarist Fathers, as they are generally called, acting through the Mother-General, who of course is the immediate head, and who resides at the central establishment of the order, the Maison Mère, at Paris. The same Superior may be elected to that office twice consecutively, but not oftener, and the tenure is for three years. The Mother-General has a council to assist her, in the persons of her assistant, the treasurer, and the dispensière, who are elected annually. Novices undergo a probation lasting five years, spending the first in spiritual training, and the remaining four in receiving instruction in the duties of their future life. The vows of poverty, obedience, and chastity are renewed annually on the Feast of the Annunciation, being the day on which the first Superior, Mademoiselle le Gras, took up her work, proving herself until her death S. Vincent's right hand in all his undertakings.

